# The prognostic capability of inflammatory proteins in predicting peripheral artery disease related adverse events

**DOI:** 10.3389/fcvm.2022.1073751

**Published:** 2022-12-13

**Authors:** Ben Li, Niousha Djahanpour, Abdelrahman Zamzam, Muzammil H. Syed, Shubha Jain, Sara Arfan, Rawand Abdin, Mohammad Qadura

**Affiliations:** ^1^Unity Health Toronto, Division of Vascular Surgery, St. Michael’s Hospital, University of Toronto, Toronto, ON, Canada; ^2^Department of Medicine, McMaster University, Hamilton, ON, Canada; ^3^Department of Surgery, University of Toronto, Toronto, ON, Canada; ^4^Unity Health Toronto, Keenan Research Centre for Biomedical Science, St. Michael’s Hospital, Li Ka Shing Knowledge Institute, University of Toronto, Toronto, ON, Canada

**Keywords:** inflammatory proteins, AGP, prognosis, peripheral artery disease, biomarker

## Abstract

**Background:**

Levels of inflammatory proteins and their prognostic potential have been inadequately studied in patients with peripheral artery disease (PAD). In this study, we quantified and assessed the ability of inflammatory proteins in predicting PAD-related adverse events.

**Methods:**

In this prospective case-control study, blood samples were collected from patients without PAD (*n* = 202) and patients with PAD (*n* = 275). The PAD cohort was stratified by disease severity based on ankle brachial index (ABI): mild (*n* = 49), moderate (*n* = 164), and severe (*n* = 62). Patients were followed for 2 years. Plasma concentrations of 5 inflammatory proteins were measured: Alpha-2-Macroglobulin (A2M), Fetuin A, Alpha-1-Acid Glycoprotein (AGP), Serum Amyloid P component (SAP), and Adipsin. The primary outcome of our study was major adverse limb event (MALE), defined as the need for vascular intervention (open or endovascular revascularization) or major amputation. The secondary outcome was worsening PAD status, defined as a drop in ABI greater than or equal to 0.15 over the study period. Multivariable logistic regression was performed to assess the prognostic value of inflammatory proteins in predicting MALE, adjusting for confounding variables.

**Results:**

Compared to patients without PAD, three inflammatory proteins were differentially expressed in patients with PAD (AGP, Fetuin A, and SAP). The primary outcome (MALE) and secondary outcome (worsening PAD) status were noted in 69 (25%) and 60 (22%) patients, respectively. PAD-related adverse events occurred more frequently in severe PAD patients. Based on our data, the inflammatory protein AGP was the most reliable predictor of primary and secondary outcomes. On multivariable analysis, there was a significant association between AGP and MALE in all PAD disease states [mild: adjusted HR 1.13 (95% CI 1.05–1.47), moderate: adjusted HR 1.23 (95% CI 1.16–1.73), severe: adjusted HR 1.37 (95% CI 1.25–1.85)]. High levels of AGP were associated with lower 2-year MALE-free survival in all PAD disease states [mild (64% vs. 100%, *p* = 0.02), moderate (64% vs. 85%, *p* = 0.02), severe (55% vs. 88%, *p* = 0.02), all PAD (62% vs. 88%, *p* = 0.01)].

**Conclusion:**

Levels of inflammatory protein AGP may help in risk stratifying PAD patients at high risk of MALE and worsening PAD status and subsequently facilitate further vascular evaluation and initiation of aggressive medical/surgical management.

## Introduction

Peripheral artery disease (PAD) primarily involves lower extremity arterial atherosclerosis, which may result in limb ischemia manifesting as claudication, rest pain, and/or tissue loss (1). Affecting over 200 million people worldwide, PAD represents a major disease burden for patients and healthcare systems (2). The gold-standard for PAD screening is the ankle brachial index (ABI) (3); however, it does not correlate well with PAD-related complications, as demonstrated by several studies (4–6). Therefore, there is a critical need to identify better prognostic biomarkers for PAD.

Previously, several proteins have been demonstrated to be associated with PAD diagnosis and disease prognosis. Syed et al. demonstrated that serum fatty acid binding protein 3 (FABP3) levels were elevated in PAD patients and associated with greater severity of ischemia (7). These findings were replicated in urinary FABP3 (8, 9). More recently, we showed that higher levels of Cystatin C correlated with long-term adverse PAD-related events (10). These results suggest that there is potential to develop a multifaceted approach to risk stratifying PAD patients using a panel of biomarkers.

Previous studies have demonstrated that inflammatory proteins contribute to cardiovascular diseases, including Alpha-2-Macroglobulin (A2M), Fetuin A, Alpha-1-Acid Glycoprotein (AGP), Serum Amyloid P component (SAP), and Adipsin (11–15). Inflammation has a central role in the development of atherosclerosis and cardiovascular risk factors including hypertension, diabetes, and dyslipidemia, all of which contribute to PAD (16–19). We hypothesize that increased levels of these inflammatory proteins may be associated with PAD-related adverse events. Herein, we assess prognostic value of various inflammatory proteins in PAD.

## Materials and methods

### Ethics approval

This study was approved by the Unity Health Toronto Research Ethics Board at the University of Toronto, Canada. All patients provided informed consent to participate in this study. Methods were carried out according to the Declaration of Helsinki (20).

### Study design and patients

We conducted a prospective case-control study, recruiting consecutive patients with and without PAD presenting to St. Michael’s Hospital vascular surgery clinics between January 2019 and March 2020. PAD was defined as an ABI less than 0.9 or toe brachial index (TBI) less than 0.7 along with one of the following clinical findings: (1) diminished pedal pulses or (2) claudication (21). Patients without PAD had an ABI ≥ 0.9 or TBI ≥ 0.7, palpable distal pulses, and no history of claudication. ABI’s and TBI’s were performed in an accredited vascular laboratory by trained technicians using automated blood pressure cuffs at the levels of the ankle and toe, respectively, and indexed to the highest brachial blood pressure. Patients with stages 3–5 chronic kidney disease [estimated glomerular filtration rate (eGFR) less than 60 mL/min/1.73 m^2^], acute or acute on chronic limb threatening ischemia, or acute coronary syndrome within the past 3 months were excluded. Additionally, patients with chronic inflammatory conditions (including autoimmune, rheumatologic, or allergic diseases) or malignancies were also excluded. These exclusion criteria were chosen because the conditions may confound serum inflammatory protein levels.

### Baseline demographic and clinical characteristics

Each patient was evaluated with a complete medical history, physical exam, ABI and TBI values, and assessment of symptomatic status related to PAD. Baseline variables recorded included age, sex, and history of hypertension [systolic blood pressure ≥ 130 mmHg, diastolic blood pressure ≥ 80 mmHg, or taking blood pressure lowering therapy (22, 23)], dyslipidemia [total cholesterol > 5.2 mmol/L, triglyceride > 1.7 mmol/L, or taking lipid lowering therapy (22, 23)], diabetes [hemoglobin A1c ≥ 6.5% or taking an antidiabetic medication (22, 23)], chronic kidney disease (eGFR less than 90 mL/min/1.73 m^2^), smoking status (non-smoker, past smoker, current smoker), coronary artery disease (abnormal cardiac stress test or coronary angiogram), congestive heart failure (symptomatic with decreased or preserved ejection fraction), and ABI (7). Definitions for cardiovascular comorbidities were based on American Heart Association guidelines (22, 23).

### Inflammatory proteins

The following inflammatory proteins were analyzed: A2M, Fetuin A, AGP, SAP, and Adipsin. These factors were chosen based on the literature demonstrating their potential relevance to patients with atherosclerosis (11–15, 24, 25). They have also not been previously studied in patients with PAD, and therefore, may act as potential prognostic biomarkers.

### Plasma sample collection and protein level quantification

Blood samples were collected from patients and inflammatory protein concentrations in plasma were measured in duplicate using MILLIPLEX MAP Human Cardiovascular Disease Magnetic Bead Panel 1 (EMD-Millipore, Billerica, Massachusetts) (26). Intra- and inter-assay coefficients of variability were < 10%, meeting the threshold for statistical acceptability (27). Prior to sample analysis, Fluidics Verification and Calibration bead kits (28) were used to calibrate the MagPix analyzer (Luminex Corp., Austin, Texas) (29). A minimum of 50 beads for each inflammatory protein was acquired using Luminex xPonent software and analyzed using Milliplex Analyst software version 5.1 (EMD-Millipore, Billerica, Massachusetts) (30).

### Follow-up and outcomes

Patients were followed up via clinic visits at 12 and 24 months. During these visits, we recorded ABI, vascular interventions, treatment changes, and adverse events. The primary outcome of our study was 2-year major adverse limb events (MALE), a composite of the need for vascular intervention (open or endovascular revascularization of lower extremity arteries) and major amputation (any lower extremity amputation above the ankle). We also investigated the individual components of MALE. The secondary outcome of our study was 2-year worsening PAD, defined as an ABI drop greater than or equal to 0.15, as previously studies have demonstrated that this threshold is clinically relevant for limb prognosis (31–33). Given that the 5 proposed proteins have been heavily investigated for cardiac and cerebrovascular events previously (12, 15, 24, 34, 35), we aimed to specifically assess their predictive value for limb- and PAD-related outcomes. To reduce the risk of confounding from cardiovascular events, we excluded patients with acute coronary syndrome within the past 3 months to ensure that our proteins were specifically associated with PAD-related adverse events.

### Statistical analysis

Based on their ABI, patients were stratified into non-PAD (ABI > 0.9), mild PAD (ABI 0.89–0.75), moderate PAD (0.74–0.50), and severe PAD (<0.50) groups. These thresholds were chosen based on the European Society for Vascular Medicine (ESVM) guidelines (36). Dividing our cohort into subgroups allowed for assessment of the association between inflammatory proteins and both the presence and severity of PAD. Furthermore, given that patients with different severities of PAD generally have different baseline characteristics, this stratified analysis reduced the risk of confounding. Baseline characteristics for each subgroup were summarized as means [standard deviations (SD)] or numbers (proportions). Differences between subgroups were calculated using one-way analysis of variance (ANOVA) followed by *post hoc* Tukey’s test for continuous variables and chi-square test for categorical variables. Inflammatory protein levels were represented as mean (SD) and compared between groups using one-way ANOVA followed by *post-hoc* Tukey’s test. Primary and secondary outcome event rates were reported for each subgroup and compared using chi-square test. Multivariable Cox proportion hazards analysis was performed to determine the relationship between inflammatory protein levels and the primary outcome of MALE in patients with mild, moderate, and severe PAD, adjusting for age, sex, smoking status, hypertension, diabetes, dyslipidemia, chronic kidney disease, congestive heart failure, and coronary artery disease. Results were presented using hazard ratios (HR) and 95% confidence intervals (CI). The cohort was then risk-stratified into patients with low and high levels of AGP, the inflammatory protein that most reliably predicted PAD-related adverse events. The threshold was the median plasma concentration of AGP in our cohort. MALE-free survival rates were summarized using Kaplan-Meier survival curves, and differences between groups was determined using log-rank test. Our sample size calculation indicated that for detection of an estimated 10% difference in the primary outcome of 2-year MALE assuming a type 1 error rate of 5% and power of 80%, 199 patients per arm would be required. Our cohort met this sample size requirement with 202 PAD patients and 275 non-PAD patients. This study will also provide a better understanding of the strength of association between the investigated proteins and PAD-related outcomes, allowing for more robust sample size calculations for future validation studies. All continuous variables had normal distribution. Statistical significance was set at a two-tailed *p* < 0.05. All analysis was carried out using SPSS software version 23 (SPSS Inc., Chicago, Illinois) (37).

## Results

### Patient characteristics

In this study, we recruited 477 patients (202 without PAD and 275 with PAD). In the PAD cohort, we stratified patients into those with mild (*n* = 49), moderate (*n* = 164), and severe (*n* = 62) disease. Compared to patients without PAD, those with PAD were older and more likely to have cardiovascular risk factors including hypertension, dyslipidemia, diabetes, chronic kidney disease (stages 1–2), coronary artery disease, congestive heart failure, and be current smokers. In addition, patients with more severe PAD were more likely to have cardiovascular comorbidities than those with mild/moderate PAD (Table 1).

**TABLE 1:** Baseline demographic and clinical characteristics.

	Non-PAD (*n* = 202)	Mild (*n* = 49)	Moderate (*n* = 164)	Severe (*n* = 62)	*P*-value
ABI range	>0.90	0.89–0.75	0.74–0.50	<0.50	
Age, mean (SD), years	62 (14)	71 (9)	69 (10)	70 (9)	0.001
Sex, male	126 (62)	32 (65)	109 (67)	42 (68)	0.811
Hypertension	99 (49)	37 (76)	122 (74)	56 (90)	0.001
Dyslipidemia	99 (49)	41 (84)	129 (79)	52 (84)	0.001
Diabetes	27 (13)	16 (33)	63 (38)	28 (45)	0.001
Chronic kidney disease	4 (2)	5 (10)	12 (7)	2 (3)	0.027
Past smoker	76 (38)	14 (29)	54 (33)	28 (45)	0.228
Current smoker	30 (15)	20 (41)	44 (27)	18 (29)	0.003
Congestive heart failure	4 (2)	3 (6)	3 (2)	6 (10)	0.011
Coronary artery disease	41 (20)	15 (31)	65 (40)	33 (53)	0.001

Values reported as *N* (%) unless otherwise specified. PAD, peripheral artery disease; ABI, ankle brachial index.

### Inflammatory protein levels are differentially expressed in patients with and without PAD

Compared to patients without PAD, those with PAD had lower mean (±SD) levels of Fetuin A (non-PAD [252.3 (73.1) pg/mL, *p* = 0.023], mild [234 (64.1) pg/mL], moderate [206 (3.03) pg/mL], severe [197 (16.7) pg/mL], *p* = 0.023) and SAP (non-PAD [10.4 (1.02) pg/mL], mild [10.6 (3.13) pg/mL], moderate [9.08 (1.19) pg/mL], severe [8.86 (8.62) pg/mL], *p* = 0.023).

Conversely, patients with PAD had higher mean (±SD) levels of AGP (non-PAD [1.77 (1.06) μg/mL], mild [1.98 (0.91) μg/mL], moderate [1.86 (0.76) μg/mL], severe [1.92 (1.31) μg/mL], *p* = 0.015). There were no differences in A2M and Adipsin levels between both groups (Table 2).

**TABLE 2:** Levels of inflammatory protein levels among patients with and without PAD.

	Non-PAD (*n* = 202)	Mild (*n* = 49)	Moderate (*n* = 164)	Severe (*n* = 62)	*P*-value
A2M, μg/mL	1.98 (1.66)	2.01 (1.28)	1.89 (0.88)	2.15 (1.04)	0.847
Fetuin A, pg/mL	252.3 (73.1)	234 (64.1)	206 (3.03)	197 (16.7)	0.023
AGP, μg/mL	1.77 (1.06)	1.98 (0.91)	1.86 (0.76)	1.92 (1.31)	0.015
SAP, pg/mL	10.4 (1.02)	10.6 (3.13)	9.08 (1.19)	8.86 (8.62)	0.023
Adipsin, pg/mL	10.51 (4.05)	13.1 (5.82)	12.9 (1.18)	12.2 (4.17)	0.391

Values reported as mean (SD). PAD, peripheral artery disease; A2M, Alpha-2-Mmacroglobulin; Fetuin A, AGP, Alpha-1-Acid Glycoprotein; SAP, Serum Amyloid P component; and Adipsin.

### Adverse events were more commonly observed in patients with PAD

Over 2 years of follow-up, all adverse events happened in PAD patients. MALE occurred in 69 (25%) patients in the following distribution based on PAD disease severity: mild [*n* = 9 (18%)], moderate [*n* = 40 (24%)], and severe [*n* = 20 (32%)]. Thirteen (4.7%) patients required major amputation (mild [*n* = 3 (6%)], moderate [*n* = 4 (2%)], severe [*n* = 6 (10%)]), 65 (23.6%) patients had vascular intervention (mild [*n* = 9 (18%)], moderate [*n* = 40 (24%)], severe [*n* = 16 (26%)]), and 60 (21.8%) patients had worsening PAD status (mild [*n* = 9 (18%)], moderate [*n* = 40 (24%)], severe [*n* = 20 (32%)]) (Table 3). As expected, adverse PAD-related events occurred most frequently in patients with severe disease.

**TABLE 3:** Adverse events.

	Non-PAD (*n* = 202)	Mild (*n* = 49)	Moderate (*n* = 164)	Severe (*n* = 62)	*P*-value
MALE	0 (0)	9 (18)	40 (24)	20 (32)	0.001
Vascular intervention	0 (0)	9 (18)	40 (24)	16 (26)	0.001
Major amputation	0 (0)	3 (6)	4 (2)	6 (10)	0.001
Worsening PAD status	0 (0)	22 (45)	29 (18)	9 (15)	0.001

Values reported as *N* (%). MALE, major adverse limb event; composite of vascular intervention and major amputation; PAD, peripheral artery disease.

### Multivariable analysis demonstrates significant association between levels of some inflammatory proteins and adverse events

**FIGURE 1:**
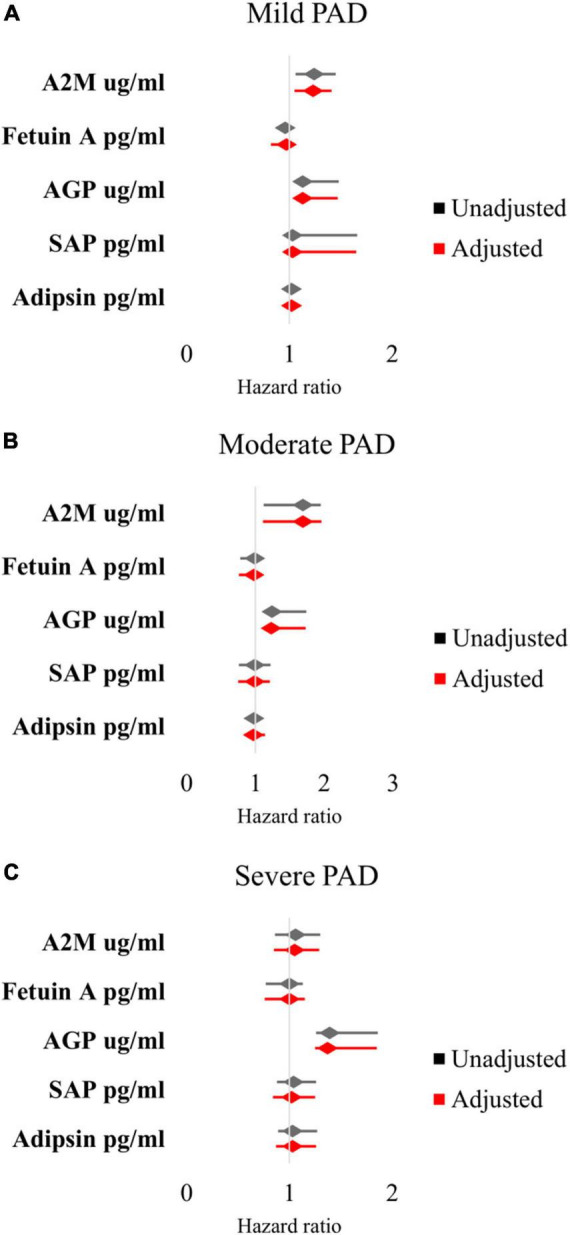
Multivariable Cox proportional hazards analysis of association between inflammatory protein levels and major adverse limb events in patients with **(A)** mild PAD, **(B)** moderate PAD, and **(C)** severe PAD. Adjusted for baseline demographic and clinical characteristics: age, sex, hypertension, dyslipidemia, diabetes, chronic kidney disease, smoking status, congestive heart failure, and coronary artery disease. PAD, peripheral artery disease.

In mild PAD patients, there was an association between MALE and A2M (HR 1.24 [95% CI 1.06–1.45], adjusted HR 1.23 [95% CI 1.05–1.41]), AGP (HR 1.13 [95% CI 1.06–1.48], adjusted HR 1.13 [95% CI 1.05–1.47]), and SAP (HR 1.03 [95% CI 1.02–1.66], adjusted HR 1.03 [95% CI 1.01–1.65]) (Figure 1A). In moderate PAD patients, there was an association between MALE and A2M (HR 1.69 [95% CI 1.12–1.95], adjusted HR 1.69 [95% CI 1.11–1.96]) and AGP (HR 1.24 [95% CI 1.17–1.74], adjusted HR 1.23 [95% CI 1.16–1.73]) (Figure 1B). In severe PAD patients, there was an association between MALE and AGP (HR 1.39 [95% CI 1.26–1.86], adjusted HR 1.37 [95% CI 1.25–1.85]) (Figure 1C).

AGP was also significantly associated with worsening PAD status in patients with mild PAD (HR 1.23 [95% CI 1.08–1.87], adjusted HR 1.22 [95% CI 1.07–1.86]). Given that AGP was the most robust predictor of primary and secondary outcomes, we risk-stratified our patients into those with low vs. high AGP levels based on the median value in our cohort.

### Risk-stratification based on AGP levels demonstrates that high levels of AGP are associated with PAD-related adverse events

**FIGURE 2:**
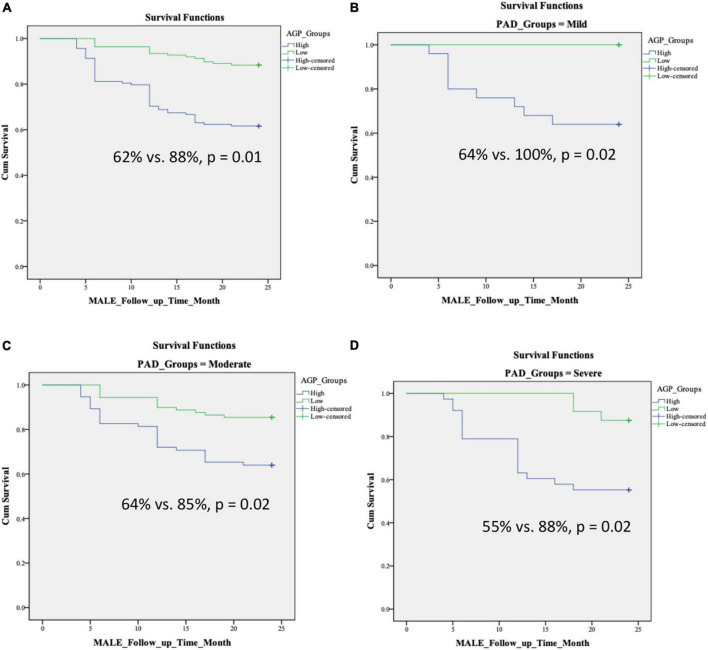
Kaplan–Meier curves demonstrating MALE-free survival rates over a 2-year follow-up period in patients with low and high AGP levels based on disease severity: **(A)** all PAD, **(B)** mild PAD, **(C)** moderate PAD, **(D)** severe PAD. Stratification into low vs. high AGP groups was based on the median level in our cohort. MALE, major adverse limb event; composite of vascular intervention and major amputation; PAD, peripheral artery disease.

**FIGURE 3:**
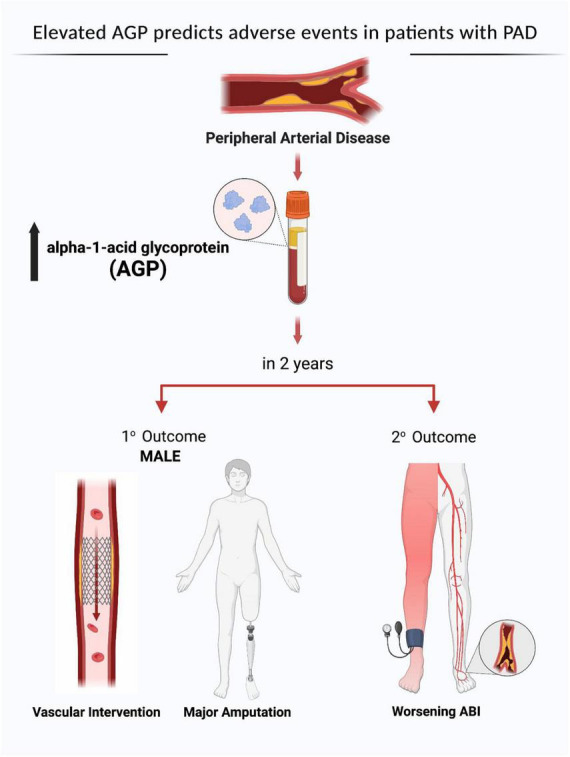
Alpha-1-acid glycoprotein (AGP) as a clinical biomarker for predicting adverse peripheral artery disease (PAD) related events. MALE, major adverse limb event; ABI, ankle brachial index. Created by the authors using BioRender.com with permission.

We found no differences in age, sex, and comorbidities including hypertension, diabetes, dyslipidemia, chronic kidney disease, coronary artery disease, congestive heart failure, and smoking status between patients with low and high levels of AGP (Table 4). Over 2 years of follow up, patients with high AGP levels had lower MALE-free survival rates [mild (64% vs. 100%, *p* = 0.02), moderate (64% vs. 85%, *p* = 0.02), severe (55% vs. 88%, *p* = 0.02), all PAD (62% vs. 88%, *p* = 0.01)] (Figure 2). In addition, patients with high AGP levels also had lower vascular intervention-free survival rates [mild (76% vs. 100%, *p* = 0.01), moderate (83% vs. 97%, *p* = 0.02), severe (71% vs. 96%, *p* = 0.02), all PAD (78% vs. 97%)], and amputation-free survival [all PAD (91% vs. 99%)]. Patients with high AGP levels had a lower freedom from worsening PAD status only in the mild disease subgroup (40% vs. 71%, *p* = 0.025), which likely represents the fact that this subgroup of patients are most likely to have significant absolute changes in ABI (e.g., patients with already low starting ABI’s are less likely to have a > 0.15 change over a 2-year period). Figure 3 summarizes the use of AGP as a clinical biomarker for predicting adverse PAD-related events.

**TABLE 4:** Baseline demographic and clinical characteristics in patients with low and high alpha-1-acid glycoprotein (AGP) levels.

	Low AGP (*n* = 238)	High AGP (*n* = 239)	*P*-value
**PAD groups**			
Mild	25 (11)	24 (10)	0.224
Moderate	75 (31)	89 (37)	
Severe	38 (16)	24 (10)	
Age, mean (SD), years	67 (12)	65 (13)	0.068
Sex, male	151 (63)	158 (66)	0.543
Hypertension	151 (63)	163 (68)	0.274
Dyslipidemia	160 (67)	161 (67)	0.975
Diabetes	62 (26)	72 (30)	0.322
Chronic kidney disease	11 (5)	12 (5)	0.839
Past smoker	81 (34)	91 (38)	0.464
Current smoker	54 (23)	58 (24)	0.684
Congestive heart failure	6 (3)	10 (4)	0.313
Coronary artery disease	75 (31)	79 (33)	0.719

Values reported as *N* (%) unless otherwise specified. PAD, peripheral artery disease.

## Discussion

In a cohort of 477 patients (202 patients without PAD and 275 patients with PAD), we demonstrated that inflammatory proteins, particularly AGP, may have good prognostic value in PAD. We showed that several inflammatory proteins are differentially expressed based on PAD disease state. Fetuin A and SAP were lower, while AGP was elevated, in PAD patients. Furthermore, we noted that AGP and A2M were correlated with MALE after controlling for baseline demographic and clinical characteristics in patients with mild and moderate PAD. AGP was the only protein that independently predicted MALE in patients with severe PAD. Furthermore, AGP was an important predictor of worsening PAD status in those with mild PAD. On stratification of our cohort based on the median AGP level, we showed that patients with higher levels of AGP had a greater risk of developing MALE and other PAD-related adverse events over a 2-year period. This is the first study demonstrating the potential of utilizing AGP as a risk stratification biomarker in PAD patients.

Inflammatory proteins have previously been studied in several disease states. We chose to assess 5 specific inflammatory proteins because they have been previously demonstrated to be associated with cardiovascular disease. Shimomura et al. showed that patients with higher A2M levels were more likely to have dyslipidemia and exhibit endothelial dysfunction (24). Jensen et al. demonstrated that Fetuin-A, a hepatic secretory protein, trended toward heightened cardiovascular risk in diabetic patients (38). In animal models of hypertensive heart disease, SAP administration was associated with unfavorable cardiac remodeling (39). Clinically, Ohtsuki et al. demonstrated that higher levels of adipsin predicted rehospitalization and mortality in patients with coronary artery disease (15). In our study, the most reliable predictor of adverse PAD-related events was AGP, which has been studied in several other contexts. Previously, Henry et al. demonstrated that AGP predicted in-hospital mortality in older patients, in addition to other cardiovascular events such as stroke and congestive heart failure (34). Elsewhere, Chu et al. showed that higher levels of AGP was correlated with myocardial infarction and congestive heart failure (35). Interestingly, Mackiewicz and Mackiewicz demonstrated the role of AGP as a marker of pro-inflammatory states and malignancy (40). The association between AGP and inflammation may explain its role in cardiovascular disease development and progression (40). Given the previously demonstrated associations between these inflammatory proteins and cardiovascular outcomes, we chose to investigate their predictive value in PAD-related limb events.

The predictive potential of classical inflammatory proteins, such as C-reactive protein (CRP), interleukins (IL) 1 and 6, and tumor necrosis factor (TNF) alpha, have been previously investigated. Singh et al. conducted a systematic review of 16 studies demonstrating an association between CRP and elevated risk of major cardiovascular events in PAD patients, with a pooled hazard ratio of 1.38 per unit increase in log_*e*_CRP (41). We demonstrated similarly strong associations between AGP and MALE, with hazard ratios ranging from 1.13 in patients with mild PAD to 1.37 in patients with severe PAD. Elsewhere, Vainas et al. demonstrated that elevated CRP was associated with lower ABI, death, and cardiovascular events in patients with PAD (42). However, the authors reported that this correlation was non-specific, as CRP was also elevated in patients with coronary plaques, aortic aneurysms, and failed coronary bypasses (42). Others have demonstrated that IL-6 and TNF-a become elevated in PAD patients after exercise treadmill testing, suggesting that inflammatory markers increase in conditions of atherosclerosis and hemodynamic stress (43). This is corroborated by Andreozzi et al. who showed that IL-1 and IL-6 were activated in claudicants and enhanced after maximal exercise (44). Others have shown that IL-1, IL-6, and TNF-a are associated with atherosclerosis in general, and therefore, may be elevated in patients with cardiac or cerebrovascular diseases (45). As a result, these markers may not be specifically associated with PAD prognosis. This is the first study assessing the prognostic value of 5 novel inflammatory markers in patients with PAD (A2M, Fetuin A, AGP, SAP, and Adipsin), which have not been previously investigated. We demonstrated that one specific inflammatory marker (AGP), has good predictive value specifically for PAD-related adverse events.

In this study, we showed that patients with more severe PAD had a greater number of risk factors and were more likely to develop MALE. This is in line with previous findings showing that patients with PAD represent a high risk population who are at increased risk of adverse events (46). Furthermore, we found that AGP was the most reliable predictor of primary and secondary outcomes in patients regardless of PAD disease severity. AGP is an acute-phase protein that contains five strongly sialylated complex glycans, making it an extremely acidic glycoprotein in plasma (47, 48). The glycans’ composition changes during inflammation, which plays a central role in the immunomodulatory properties of AGP (49). Although the exact physiological function of AGP remains an area of heavy investigation, it plays a role in dampening excessive inflammatory reactions (49). AGP is therefore a marker of inflammation, which has been demonstrated to play an essential role in PAD (43). A pro-inflammatory state contributes to atherosclerosis by promoting the buildup of low-density lipoproteins (LDL) in the intima and stimulation of endothelial dysfunction (50). Given that atherosclerosis forms the basis of PAD and its complications, elevated AGP levels may therefore act as a pathological biomarker for PAD. This was highlighted in our study when we showed elevated AGP levels in patients with PAD. Lastly, we were able to demonstrate the prognostic value of AGP in PAD by risk-stratifying our cohort into patients with low vs. high AGP levels. We demonstrated that individuals with higher AGP levels had a greater risk of developing PAD-related adverse events over a 2-year period. This suggests that PAD patients with higher baseline levels of inflammation, as measured by AGP, are more likely to have limb-related complications over the long term. Our paper provides some evidence that AGP can be used as a potential prognostication biomarker for PAD, adding to our repository of potential biomarkers to improve prognosis of this condition via a multifaceted approach (7–10).

This study has limitations that warrant mentioning. First, all patients were recruited from a single center, thus potentially affecting generalizability of our results. Second, while we reported outcomes over a 2-year period, a longer follow-up may have proved more informative with regards to understanding the prognostic value of AGP. Third, we excluded patients with certain disease conditions. Therefore, our results may not apply to all PAD patients. Additional studies are required to validate the potential of AGP in patients with PAD. Fourth, we did not analyze other potential inflammatory markers such as CRP, IL-1/6, and TNF-a due to risk of non-specificity for PAD prognosis (42, 45); however, further investigation may be warranted. Fifth, our study focused on limb-related outcomes relevant to PAD patients. Cardiac and cerebrovascular events were not included as endpoints; however, it may be valuable to assess these outcomes in future studies.

## Conclusion

In this study, we showed that AGP, an inflammatory protein, has prognostic value in PAD. Higher AGP levels are independently associated with the development of adverse PAD-related events, including MALE. Measurement of AGP may guide risk-stratification strategies by identifying individuals at high risk of developing PAD-related limb complications. These patients may benefit from further clinical assessment, close follow-up, and aggressive medical/surgical management. Larger studies with longer-term follow-up are needed to confirm the findings in our study.

## Data availability statement

The original contributions presented in this study are included in the article/supplementary material, further inquiries can be directed to the corresponding author.

## Ethics statement

The studies involving human participants were reviewed and approved by the Unity Health Toronto, University of Toronto, Canada. The patients/participants provided their written informed consent to participate in this study.

## Author contributions

SJ, ND, MS, and SA: acquisition, analysis and interpretation of data, revising the manuscript for important intellectual content, and approval of the final manuscript draft submitted for publication. BL, ND, and AZ: methodology, statistical analysis, data analysis and interpretation, writing—original draft, revising the manuscript for important intellectual content, and approval of the final manuscript draft submitted for publication. RA and MQ: study concept and design, methodology, data analysis and interpretation, writing—original draft, revising the manuscript for important intellectual content, and approval of the final manuscript draft submitted for publication. All authors reviewed the manuscript and agreed to be accountable for all aspects of the work, ensuring the accuracy and integrity of the publication.
